# Waardenburg syndrome type 2 with a *de novo* variant of the *SOX10* gene: a case report

**DOI:** 10.1186/s12920-024-01877-9

**Published:** 2024-04-24

**Authors:** Yuanyuan Li, Yuxue Chen, Yang Sun, Shouxin Li, Lingli Dong, Zongzhe Li, Guifen Shen

**Affiliations:** 1 Department of Rheumatology and Immunology, Tongji Hospital, Tongji Medical College, Huazhong University of Science and Technology, 1095 Jiefang Avenue, 430030 Wuhan, Hubei P.R. China; 2 Division of Cardiology, Department of Internal Medicine and Genetic Diagnosis Center, Tongji Hospital, Tongji Medical College, Huazhong University of Science and Technology, 430030 Wuhan, Hubei P.R. China; 3 Hubei Key Laboratory of Genetics and Molecular Mechanisms of Cardiological Disorders, Huazhong University of Science and Technology, 430030 Wuhan, Hubei P.R. China

**Keywords:** Waardenburg syndrome type 2, Systemic lupus erythematosus, Abnormal sexual development, Whole-exome sequencing, Sex-determining region Y-box containing gene 10

## Abstract

**Background:**

Waardenburg syndrome type 2 (WS2) has been reported to be a rare hereditary disorder, which is distinguished by vivid blue eyes, varying degrees of hearing impairment, and abnormal pigment deposition in the skin and hair. Variants in the sex-determining region Y-box containing gene 10 (*SOXl0*) gene may cause congenital deafness and have been demonstrated to be important during the development of WS2.

**Methods:**

Complete clinical data of the proband and her family members (her parents and 2 sisters) was collected and physical examinations were performed in the hospital. The laboratory examination including hemoglobin, Coomb’s test, urine protein, ENA, autoimmune hepatitis-related autoantibodies and ultrasonography were all conducted. We obtained the peripheral blood samples from all the participants and performed whole exome sequencing and sanger sequencing validation.

**Results:**

The present study identified a family of 5 members, and only the proband exhibited typical WS2. Beyond the characteristics of WS2, the proband also manifested absence of puberty. The proband and her younger sister manifested systemic lupus erythematosus (SLE). Whole exome sequencing revealed a de novo variant in the *SOX10* gene. The variant c.175 C > T was located in exon 2 of the *SOX10* gene, which is anticipated to result in early termination of protein translation.

**Conclusion:**

The present study is the first to report a case of both WS2 and SLE, and the present findings may provide a new insight into WS2.

**Supplementary Information:**

The online version contains supplementary material available at 10.1186/s12920-024-01877-9.

## Introduction

A rare hereditary condition called Waardenburg syndrome (WS) mainly causes following typical symptoms: accumulated pigment in the skin, eyes and hair, various degrees of sensorineural hearing impairment [1]. There are four subtypes of the typical syndrome (WS1-4) based on their additional symptoms except for the typical symptoms above. The type I of WS is characterized by dystopia canthorum. (WS1, OMIM193500). There are no additional symptoms in WS type 2 (WS2, OMIM 193,510, 600,193, 606,662, 608,890 and 611,584). The type III of WS represents dystopia canthorum and aberrant musculoskeletal function of the upper extremities (WS3, OMIM148820). The type IV of WS is featured with Hirschsprung Disease (WS4, OMIM 277,580, 613,265 and 613,266). Variable phenotype of WS can be observed in both interfamily and intrafamily, suggesting that there is an interplay between genetic modifiers and environmental factors [2].

WS is genetically heterogeneous. Several genes are involved in the pathogenetic process of WS, such as melanocyte inducing tyrosinase related protein 1, transcription factor (MITF), endothelin (EDN) 3, paired box 3 (PAX3) transcription factor, EDN receptor type B (EDNRB), Snail homolog 2 (SNAI2) and sex-determining region Y-box containing gene 10 (SOX10), accompanied with varying frequencies [3]. Approximately 15% of WS2 cases are caused by *SOX10* variants [4, 5]. In 2002, two patients diagnosed with WS and *SOX10* variants were reported to exhibit semicircular canals agenesis for the first time [6].

The *SOX10* gene is composed of five exons, and it belongs to the SOX family of transcription factors. The typical gene was also reported to determine the cell fate and to participate in the development of cell lineage, which plays a significant role in the early developmental stages of the peripheral nervous system and neural crest. The *SOX10* protein contains a DNA-binding domain named high mobility group (HMG), which is highly conserved and consists of 80 amino acids [5]. *SOX10* was also reported to regulate embryonic development [7].

The present report describes a 28-year-old female with bilateral hearing damage, pigmentation abnormalities and hypogonadism. Her condition was considered to be a clinical phenotype 2 of WS with a rare *SOX10* variant complicated with systemic lupus erythematosus (SLE). The purpose of the study was to report this case for its rarity and to review the relevant literature.

## Case description

Complete clinical data of the patient and her family members (her parents and two sisters) were collected, and physical examinations were performed at the Tongji Hospital (1095 Jiefang Avenue, Wuhan, Hubei). We performed experiments according to the Declaration of Helsinki. Consent was obtained from each participant enrolled in our study.

**Fig. 1:**
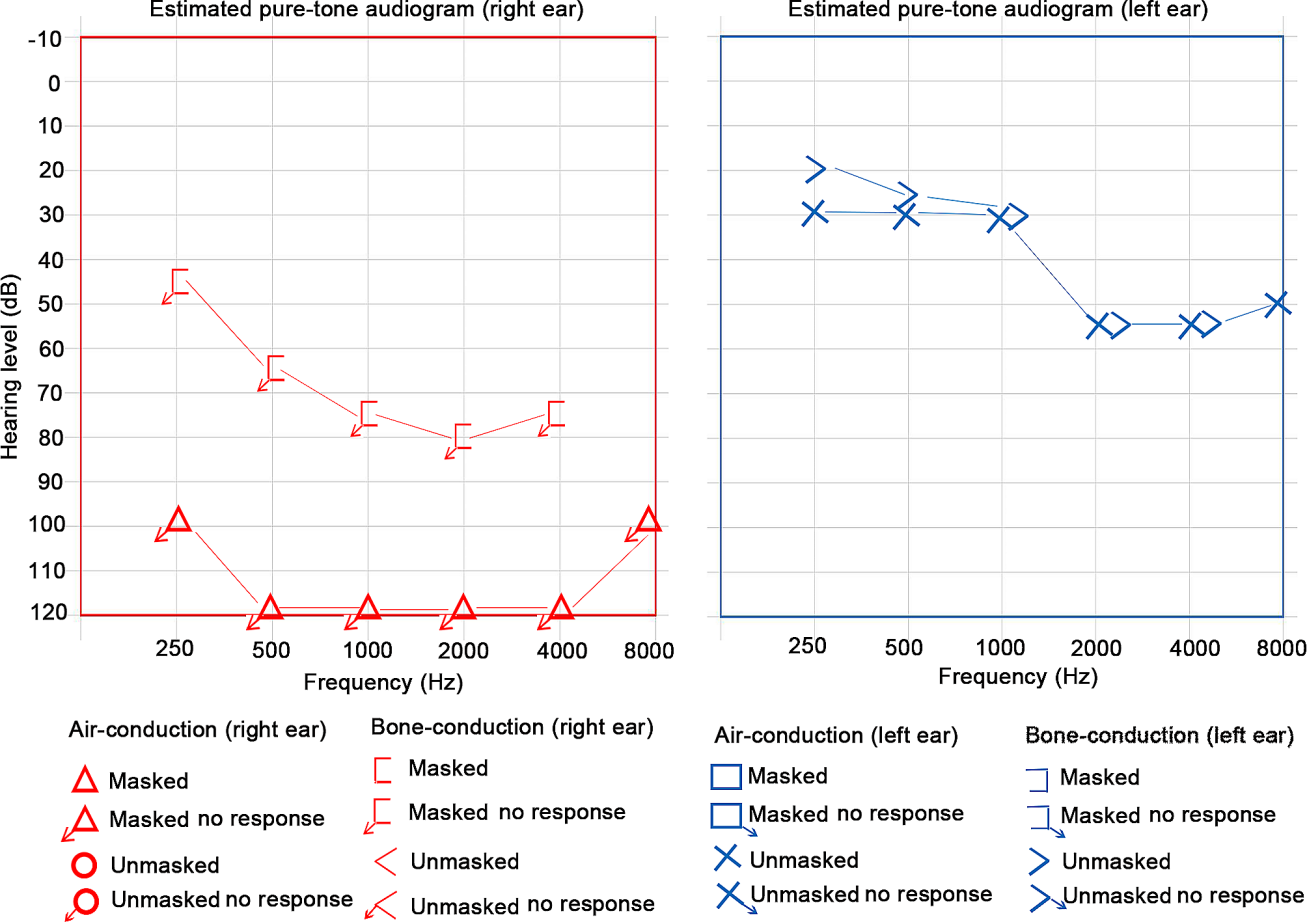
Multi-frequency stimuli based pure-tone audiogram demonstrated bilateral hearing loss (right ear > 100 dB hearing level, left ear > 30 dB hearing level) and left sensorineural hearing loss of the proband

A 28-year-old Chinese woman presented to our hospital for further diagnosis and therapy on June 30, 2020. She had a 6-month history of polyarticular pain, which mainly covered bilateral shoulders, wrist, knee, hip, elbow and proximal interphalangeal joints. Meanwhile, she noted with hair loss, oral ulcer, xerostomia and Raynaud’s phenomenon, but denied morning stiffness, fever, cough, expectoration or chest pain during the course of the disease. Upon physical examination, she was found to have hearing loss of the left ear, irises in blue color and premature grayed hair. Estimated pure-tone audiogram was conducted in our hospital, which indicated bilateral hearing loss (right ear > 100 dB hearing level, left ear > 30 dB hearing level) and left sensorineural hearing loss of the proband (Fig. 1). Further investigation of her medical history revealed that she had been diagnosed with primary amenorrhea and exhibited no signs of pubertal sex development. The whole medical history of her family was gathered, and physical examinations were performed on all the family members. Environmental factors were not considered as a possibility.

Through the clinical manifestations above, the woman presented joint, skin and mucous membrane involvement. According to the findings, the patient can be inferred as connective tissue disease (CTD). But her hearing loss, blue irises, premature grayed hair and delayed sex development could not be elucidated by CTD. Then we conducted laboratory examinations for further exploration.

**Fig. 2:**
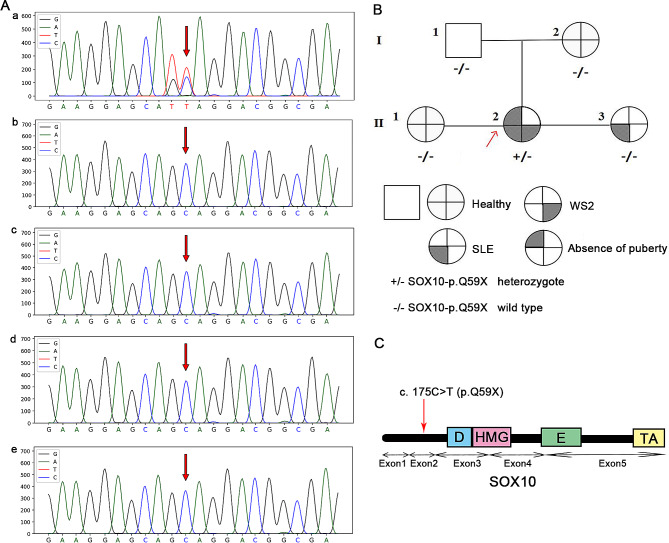
Identification of a *de novo* variant in the *SOX10* gene [transcript ID: NM_006941.3; variant c. 175 C > T (p. Q59X)]. (A) Sanger sequencing of the family. (a) The proband was identified to have the novel variant, while her (b) father, (c) mother and (d and e) two sisters exhibited the wild-type genotype. (B) Family tree of the Waardenburg syndrome type 2 pedigree. The arrow indicates the proband. +/- represents the heterozygous *SOX10*-p. Q59X variant. -/- represents wild-type. (C) Affected region of the novel variant. HMG, high mobility group DNA-binding domain; D, dimerization domain; E, conserved domain of *SOX10*; TA, transactivation domain; SOX10, sex-determining region Y-box containing gene 10

Laboratory examinations, including hemoglobin levels, Coombs test, protein in urine, extractable nuclear antigen(ENA), autoimmune hepatitis-related autoantibodies and ultrasonography, were all conducted in Tongji Hospital (1095 Jiefang Avenue, Wuhan, Hubei). Peripheral blood samples were obtained from all the participants. The expression of adrenocorticotropic hormone (ACTH) was below 5.0 pg/ml. The level of 17α-hydroxyprogesterone was 0.03 nmol/l and of androstenedione was 0.21 nmol/l, which were both below the lower limit of the reference interval (Table SI). Gynecology ultrasonography showed infantile uterus and endometrial cavity fluid. Hemoglobin was moderately reduced and Coombs test was positive. Urinary protein was positive and globulin was slightly elevated. ENA showed antinuclear antibodies (ANA) 1:3,200, which is a nuclear particle type. In addition, anti double-stranded deoxyribonucleic acid (anti-dsDNA), anti ribonucleoprotein (anti-RNP), anti-Smith, anti-Ro/SSA antibodies were positive. Autoimmune hepatitis-related autoantibodies, including anti-mitochondrial M2, anti-GP210 and anti-Ro-52 antibodies, were positive. Erythrocyte sedimentation rate (ESR) (77 mm/h) and IgG (32.08 g/l) were increased. Based on her clinical symptoms and laboratory examination, the patient was diagnosed with SLE. After prednisone (40 mg, qd) and mycophenolate mofetil (0.5 g, bid) standard therapy, the SLE of the patient was kept in a stable condition. Then the prednisone gradually decreased to 7.5 mg (qd) to avoid the side effects. The intervention adherence and tolerability were assessed by testing hemogram, hepatic function, renal function, urine routine once a month and all were kept well. During the course of disease, there existed no adverse and unanticipated events. Timeline of the medical diagnosis and treatment process had been shown in Fig. 2, 3. Unexpectedly, her younger sister also had a history of SLE, whereas no other abnormal clinical manifestations were found in her father, her mother and her older sister.

**Fig. 3:**
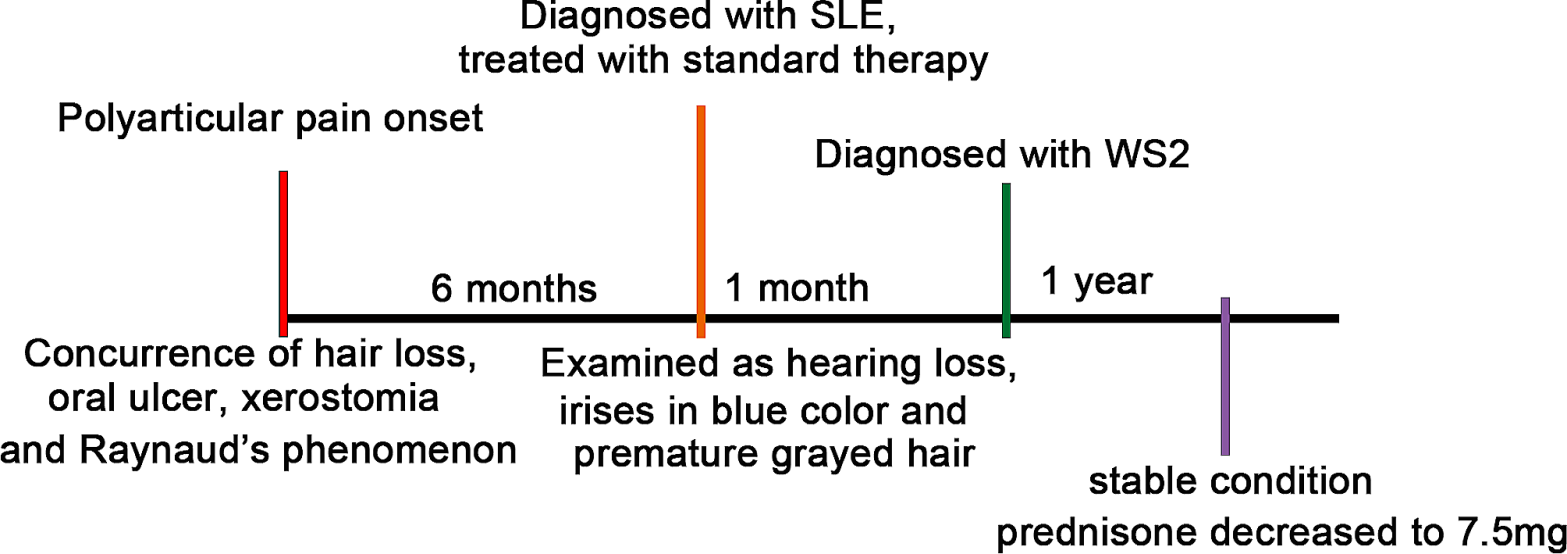
Timeline of the medical diagnosis and treatment process

### Genetic analysis

Apart from the SLE manifestation, the additional symptoms such as hearing loss of the left ear, irises in blue color and premature grayed hair can not be explained. Then the genetic analyses were performed on our patient as a part of diagnostic procedure.

Firstly, we constructed the deoxyribonucleic acid (DNA) library. The extraction and purification of the genomic DNA in this family were performed with a DNA extraction kit (Tiangen Biotech Co., Ltd.) and Tiangen Automatic Nucleic Acid Extractor (Tiangen Biotech Co., Ltd.). µThe DNA purity was detected with Nanodrop One (NanoDrop Technologies; Thermo Fisher Scientific, Inc.). The absorbance (A)260/280 and A260/230 values were 1.6-2.0. After purification, 150 ng genomic DNA was employed for enzymolysis by fragmentation enzyme. End repair and ‘A-tail’ adding were then conducted. The fragment length was 250–300 bp. Briefly, the following steps included splice ligation, pre-PCR and library purification, library quantification and pooling, concentration, hybrid capture, enrichment and recovery of the target area, post-PCR amplification and purification, library quantification and quality assessment. XGen™ Exome Research Panel v1 (Integrated DNA Technologies, Inc.), HiFi Ready Mix Enzyme (Kapa Biosystems; Roche Diagnostics) and other library construction reagents (Vazyme Biotech Co., Ltd.) were used. The final concentration of the library was evaluated by Qubit 4.0 (Thermo Fisher Scientific, Inc.) and Qubit™ dsDNA HS Assay Kit (concentration > 10 ng/µl). Library fragment length (300–550 bp) was detected using QSeq400 Fragment Analyzer (BIOptic Inc.).

Secondly, the synthetic DNA library was used for whole exome sequencing. µµµWhole exome sequencing was performed using the NovaSeq 6000 platform, NovaSeq 6000 S4 Reagent Kit (Illumina, Inc.) and S4 flowcell with PE150 (read length 150 bp) mode. Point variants and small fragment deletion/insertion variants within 20 bp were detected. The raw BCL data were converted to FASTQ format, and then annotated and analyzed.

Thirdly, we conducted the bioinformatics analysis with the raw data. All data were filtered by fastp software (HaploX Inc.), compared with the hg19/GRCh37 human reference genome by using the bwa software, and corrected by public dbsnp159 database. Only on-target reads were used to call variants using gatk 3.8. Sequencing depth was detected by samtools depth. Statistics of bed file region coverage and 20X sequencing depth was conducted by gatk depth of coverage. Mosdepth 0.2.5 software was used for GC content statistics. The reported mutations were detected using the Clinical Significance for Variants Relative to Phenotypes (ClinVar) (https://www.ncbi.nlm.nih.gov/clinvar/) and human gene mutation database (HGMD) (http://www.hgmd.cf.ac.uk/ac/search.php) database. The data that covered < 20-fold and variants whose minimum allele frequency (MAF) was > 0.01% in the Exome Aggregation Consortium database were removed. Next, the noncoding and synonymous variants were removed. Furthermore, the potential pathogenic missense variants were identified by the Polyphen-2 (score > 0.85) (http://genetics.bwh.harvard.edu/pph2/) and sorts intolerant from tolerant (SIFT) (score < 0.05) (http://sift.jcvi.org). Point variants were annotated using ANNOVAR software, and further annotation was analyzed with local databases such as HGMD, human phenotype ontology (HPO) and internal carryover rate. The pathogenic variant was referred to the American College of Medical Genetics and Genomics (ACMG) grading criteria [4].

The whole exome sequencing above was performed for screening possible variants of genes associated with metabolic, endocrine and immune diseases. A total of 1,397 known or novel variations were detected by semiconductor sequencing (Table SII). A heterozygous nonsense variant of *SOX10* (Fig. 2), c. 175 C > T (p. Q59X), was predicted to be pathogenic according to the ACMG grading criteria. The *SOX10* variant has an effect on hearing and pigmentation [5], which was considered to be the cause of WS2 in the patient. The diagnosis of WS2 was made on the basis of the clinical manifestations above and the international criteria raised by the Waardenburg Consortium [7]. No other variants related to WS were identified in the patient.

Furthermore, the sanger sequencing validation of the *SOX10* gene was then performed with all the relatives of the patient. After enzymatic lysis, the PCR products were labeled with fluorescence signals via the terminal dideoxygenation method for capillary electrophoresis separation [6]. The forward primer of the *SOX10* gene is 5’-GTTGGACTCTTTGCGAGGAC-3’ and the reverse primer is 5’-TGACGTGCGGCTTGCTTTTG-3’. PCR amplifications were performed with an ABI 9700 (Thermo Fisher Scientific, Inc.) as follows: denaturation of 96˚C for 3 min, followed by 30 cycles of denaturation at 96˚C for 15 s, annealing at 56˚C for 15 s and extension at 60˚C for 1 min, and a final extension of 72˚C for 5 min. The purified PCR products with a size of a 391 bp were sequenced by Sanger sequencing. The result showed that *SOX10* variant in the patient does not exist in her family members and it was considered to be a *de novo* variant. In the meantime, no other *SOX10* variants were identified in other family members.

Finally, the proband was diagnosed with WS2 containing a rare *SOX10* heterozygous variant and she suffered from SLE simultaneously.

## Discussion

The present study reports a Chinese woman diagnosed with WS2, who had a c. 175 C > T (p. Q59X) heterozygous variant in exon 2 of the *SOX10* gene. The patient manifested auditory malfunction of both ears, blue-colored irises, premature graying of the hair which are characteristics of WS2. In addition, the patient was absent of pubertal sex development, and exhibited primary amenorrhea, low gonadal hormone, infantile uterus and breast dysplasia. The additional manifestation may be explained by the fact that distinct categories of variants in the *SOX10* gene may display various clinical features by affecting different signaling pathways in WS. It has been reported that *SOX10* gene variants may result in pubertal delay [8, 9]. One case was identified as co-existence of WS2 and Kallmann syndrome associated with *SOX10* gene variants [10]. Meanwhile, in the present study, no other gene variants were associated with the absence of pubertal sex development.

The ACTH level of the patient was under the lower limit of detection. ACTH has been reported to be important in the regulation of melanocytes [11]. ACTH plays a crucial role in melanocyte differentiation and melanogenesis regulation through the cAMP signaling pathway. It has been suggested that melanogenesis upregulation is associated with cAMP [12]. In the present study, *SOX10* gene variant and low level of ACTH were detected. Thus, it was speculated that *SOX10* gene variants may cause melanocyte dysfunction through downregulating the expression of ACTH. However, further research on the pathogenesis of WS is required in the future.

Notably, the patient also presented SLE characteristics, including polyarticular pain, hair loss, oral ulcer, xerostomia and Raynaud’s phenomenon. To date, no cases diagnosed with WS and SLE have been reported. Besides, the family may have a genetic predisposition to SLE. But no studies have been reported the association between the predisposition to SLE and *SOX10* gene. The coexistence of WS and SLE may be a coincidence, and it was also speculated that other gene variants may exist in the proband and one of her sisters. As previously reported, there are various genes linked to monogenic lupus [13, 14]. Through searching these genes in the proband, gene variants of interferon stimulated gene 15 (ISG15), ribonuclease H2, subunit B (RNASEH2B) and SOS Ras/Rac guanine nucleotide exchange factor 1 (SOS1) were found in the proband, and the dysfunction of these genes may be related to SLE pathogenesis in the proband. Thus, it was speculated that, in a patient with WS, this may combine with any genetic or non-hereditary disease.

The c. 175 C > T (p. Q59X) variant was detected as a heterozygous variant located in the second exon of *SOX10*. After analyzing her family members, only the proband contained the variant, indicating that it was a *de novo* pathogenic variant. The variant of this type was evaluated as pathogenic, and this new variant was absent in HGMD and Clinvar databases. No other WS associated gene (such as MITF, PAX3 or SNAI2) variants were identified. The current study is the first to report the c. 175 C > T (p. Q59X) variant in the *SOX10* gene. The truncating heterozygous variant of the *SOX10* gene in the present study is a *de novo* pathogenic variant which caused WS2 associated manifestation in a Chinese woman. Previous studies have shown that ∼ 15% of WS2 pathogenesis is related to variants in the *SOX10* gene, and several *SOX10* gene heterozygous variants have been reported in WS2 in Chinese family [15–18]. The present findings expand the knowledge of *SOX10* gene variants in WS2.

The *SOX10* gene is situated on chromosome 22q 13.1 and contains five exons. Among the five exons, only exons 3, 4 and 5 encode protein. The *SOX10* protein consists of 466 amino acids. It has a size of 51 kDa and belongs to the *SOX* gene family [19]. *SOX10* contains a central DNA-binding domain named high mobility group (HMG) domain, which is a highly conserved and active domain, and a C-terminal transactivation domain [20]. The HMG domain can specifically identify and bind to the promoters of the target genes, which allows *SOX10* to act as a key transcription factor during the process of migration and differentiation of neural crest cell, including melanocytes, olfactory ensheathing cells, intestinal ganglion cells, and numerous sensory and autonomic ganglion cells [21–23]. The transactivation domain of *SOX10* activates the transcription of the MITF, tyrosinase and EDNRB genes [24]. The target genes of *SOX10* (including MITF, tyrosinase and EDNRB) participate in melanin synthesis [21, 22]. SOXl0 plays a crucial role in the pathogenesis of WS, and SOXl0 variants may lead to WS2 and WS4, which can result in hearing impairment and pigmentary abnormalities of the hair, iris and skin [15]. In addition, variants in *SOX10* may cause other neural crest-related diseases, including central demyelinating leukodystrophy, peripheral demyelinating neuropathy and Hirschsprung disease [25, 26].

The present study identified a *de novo* variant in the *SOX10* gene, which provides a new perspective into the mechanism of WS and contributes to updating the HGMD and Clinvar databases. Moreover, the current study is the first to report a case of WS2 complicated with SLE, and the present findings may lay a vital foundation for non-invasive prenatal genetic diagnosis and genetic counseling. Furthermore, the co-occurrence of WS2 and SLE in the current case broadens the recognition that a patient with WS may combine any genetic or non-hereditary disease, which may contribute to the field of disease diagnosis in the future. The association between WS2 and SLE should be further explored. In addition, targeted gene therapy should be emphasized for *SOX10* and other genes in patients with WS2. Moreover, it is necessary to pay particular attention to the sexual development of patients with WS2 with *SOX10* variants during adolescence.

### Electronic supplementary material

Below is the link to the electronic supplementary material.

Supplementary Material 1

Supplementary Material 2

## Data Availability

All data generated or analyzed during the present study are included in this published article.
