# Supplementary material for: Waardenburg syndrome type 2 with a de novo variant of the SOX10 gene: a case report
Source: BMC Med Genomics. 2024 Apr 24;17:104. doi: 10.1186/s12920-024-01877-9 (PMC11040914; doi:10.1186/s12920-024-01877-9)
Supplement: Supplementary file 1 — Supplementary Material 1 [file 12920_2024_1877_MOESM1_ESM.pdf]

Table SI Sex development-related indicator of the proband

Sex development-related indicator			
Sex development-related indicator	Result	Reference Interval	units
Progestone	0.15	Mid follicular phase: 0.31-1.52; Mid luteal phase: 5.16-18.56; Menopause: 0.00-0.78	ng/mL
Follicle stimulating hormone	2.01	Follicle stage: 3.85-8.78 Ovulatory stage: 4.54-22.51 Luteal stage: 1.79-5.12 Menopause: 16.74-113.59	mIU/mL
Luteinizing hormone	0.4	Follicle stage: 2.12-10.89 Ovulatory stage: 19.18-103.03 Luteal stage: 1.2-12.86 Menopause: 10.87-58.64	mIU/mL
Estrogen	<20	Follicle stage: 27-122 Ovulatory stage: 95-433 Luteal stage: 49-291 Menopause: ⩽40	pg/mL
Testosterone	<0.1	⩽0.75	pg/mL
Prolactin	8.51	Premenopause: 3.34-26.72 Postmenopause: 2.74-19.64	ng/mL
Menstruation	Primary amenorrhea	—	—
Uterine	Infantile uterus	—	—
Breast	Undeveloped breasts	—	—
